# Flower Volatiles, Crop Varieties and Bee Responses

**DOI:** 10.1371/journal.pone.0072724

**Published:** 2013-08-20

**Authors:** Björn K. Klatt, Carina Burmeister, Catrin Westphal, Teja Tscharntke, Maximillian von Fragstein

**Affiliations:** 1 Agroecology, University of Göttingen, Göttingen, Germany; 2 Centre for Environmental and Climate Research, University of Lund, Lund, Schweden; 3 Buesgen-Institute University of Göttingen, Göttingen, Germany; French National Institute for Agricultural Research (INRA), France

## Abstract

Pollination contributes to an estimated one third of global food production, through both the improvement of the yield and the quality of crops. Volatile compounds emitted by crop flowers mediate plant-pollinator interactions, but differences between crop varieties are still little explored. We investigated whether the visitation of crop flowers is determined by variety-specific flower volatiles using strawberry varieties (*Fragaria x ananassa* Duchesne) and how this affects the pollination services of the wild bee *Osmia bicornis* L. Flower volatile compounds of three strawberry varieties were measured via headspace collection. Gas chromatography showed that the three strawberry varieties produced the same volatile compounds but with quantitative differences of the total amount of volatiles and between distinct compounds. Electroantennographic recordings showed that inexperienced females of *Osmia bicornis* had higher antennal responses to all volatile compounds than to controls of air and paraffin oil, however responses differed between compounds. The variety Sonata was found to emit a total higher level of volatiles and also higher levels of most of the compounds that evoked antennal responses compared with the other varieties Honeoye and Darselect. Sonata also received more flower visits from *Osmia bicornis* females under field conditions, compared with Honeoye. Our results suggest that differences in the emission of flower volatile compounds among strawberry varieties mediate their attractiveness to females of *Osmia bicornis*. Since quality and quantity of marketable fruits depend on optimal pollination, a better understanding of the role of flower volatiles in crop production is required and should be considered more closely in crop-variety breeding.

## Introduction

The global increase in food demand, due to a rapidly rising world population [1], highlights the importance of world food security [2]. Pollination contributes to more than one third of crop yield worldwide [3], and appears to be a key factor in maintaining the stability of agricultural food production [4]. However, our knowledge about crop pollination is still limited [5]. Declining pollinator populations are threatening pollination services [6] and highlight the need to expand the knowledge-base of bee-flower interactions in order to maintain pollination services [7].

The influence of floral traits, such as colour, shape and structure, on the attraction of bees has been widely studied [8]. But floral scent can also have a great influence on attracting pollinators, and flower volatile compounds have been suggested as the main drivers for visitation decisions by pollinators, including flower constancy [7], [9], [10]. However, these studies have focused mainly on honeybees, bumble bees [10] and a few specialised wild bee species [7]. Thus relationships between pollination ecology and chemical ecology are still weakly studied [7], [11] and in particular our knowledge about how crop varieties attract pollinators remains scarce [12], [13]. The concentration of flower volatile compounds can vary due to genetic differences among subspecies [14] and plant populations at different locations [15]. Only a few studies to date have reported on differences between crop varieties [12], [16], [17], [18], [19], [20], [21]. Of these, only four studies investigated the influence on pollinator attraction of varieties differing in volatile emissions [12], [16], [17], [19], but mainly focused on honeybees.

In the current study we aim to highlight the importance of volatile emissions for the attractiveness of crop varieties to wild bee pollinators. Wild bees have been found to be important crop pollinators [22] that can be even more efficient than honeybees for various reasons as better performance in pollen exchange, transfer and deposition as well as interspecific interactions with honeybees [23]. Recent declines of honeybees [24], [25] further demonstrate the importance of pollinaton services offered by wild bees for the future, but clearly less is known about the general mechanisms of attraction for solitary wild bees [7]. Mason bees (*Osmia* spp.) have been identified as providing important crop pollination services [23], [26] and have been shown to be a suitable pollinator for strawberries [27]. The foraging behaviour of the red mason bee *Osmia bicornis* L. has recently been shown to be strongly influenced by floral scent [28], however details about distinct compounds involved have not been presented to date.

Strawberries benefit from pollination through enhanced fruit shape and weight [29], [30]. Strawberry breeding focuses on several plant parameters, which differ between varieties, such as sensitivity to fungal infections and diseases, harvest time and taste [31], but attractiveness to pollinators appears to be neglected. Volatile composition and quantities have, to date, been tested for a single variety of commercial strawberries [32], and female and hermaphroditic flowers of wild strawberry (*Fragaria virginiana*) are known to differ in their emission of volatiles [33]. Differences in floral volatile emission among strawberry varieties and their influence on pollinators are still unknown.

Here we analysed (i) the emission of flower volatile compounds, comparing three simultaneously flowering strawberry varieties, (ii) the antennal response of females of *O. bicornis* to these compounds and (iii) differences in the abundance of *O. bicornis* females on a commercial strawberry field. We expected strawberry varieties to differ in the qualitative and quantitative emission of flower volatile compounds. We also predicetd that antennal responses of *O. bicornis* females would differ between compounds and that this differing response would mediate the visitation rates of *O. bicornis* females between strawberry varieties under field conditions.

## Methods

Farmers contributing to this study were informed and permits were obtained prior to conducting the study. Refrigerated strawberry plants of the simultaneously flowering varieties Sonata, Honeoye and Darselect (*Fragaria x ananassa* Duchesne) were grown separately in 10 litre vessels in controlled conditions (20°C; 60% RH; 12 h daylight per 24 h), to control for variation in volatiles due to environment conditions [34]. Volatiles were sampled simultaneously on all plants. Varieties differed in the amount of open flowers (F_2,19_ = 5.278; p = 0.015; n = 22), but produced similar total mass of flowers (F_2,19_ = 0.839; p = 0.448; n = 22).

Volatiles were sampled, directly from flowers, on a charcoal trap (CLSA-Filter, Daumazan sur Arize, France) using a modified push-pull headspace collection system [35]. The flowers were enclosed in a plastic “roasting bag” (Melitta GmbH, Minden, Germany). Air was circulated through the trap by a miniature pump (Fürgut, Aichstetten, Germany) at a flow rate of 0.8 l min^−1^. The sampling time was 2 hours. Adsorbed volatiles were eluted with 50 µl of dichloromethane/methanol (2∶1). The solvents used were of analytical quality (Suprasolv quality, Merck/VWR, Darmstadt, Germany). After elution, samples were stored in an ultralow temperature freezer at −80°C.

Volatile samples were analysed with a coupled gas chromatography–mass spectrometry (GC-MS) consisting of a gas chromatograph Agilent type 6890 connected to a type 5973 quadrupole mass spectrometer (both Palo Alto, USA) with electron ionisation (EI, 70 eV). Two column types in a similar setup, a HP-5 ms (Agilent, 30 m, 0.25 mm ID, and 0.25 µm film thickness, phenylmethylsiloxane), and a HP-INNOWax (Agilent, 30 m, 0.25 mm ID, and 0.25 µm film thickness, polyethylenglycol), were used to analyse the composition of the extracts. An aliquot of 1 µL was injected into the injector held at 250°C. The oven temperature program was 50°C held for 1.5 min, followed by an increase of 7.50°C/min to 200°C, remaining at 200°C for 5 min. Helium (purity 99.999%) was used as carrier gas (1 ml/min).

For identification of the constituents, mass spectra GC retention values and linear retention indices [36] were compared to those of authentic standards and those of the mass spectral databases and published parameters (Table 1, 2). Databases used, were Wiley 9 combined with NIST ´08 [37] and “Terpenoids and Related Constituents of Essential Oils”, a database available from MassFinder 3.07 software (Hochmuth Scientific Consulting, Hamburg, Germany).

**Table 1:** Identified flower volatile compounds of three strawberry varieties (ng g^−1^ flowers). Total amounts and aldehydes.

			Darselect		Honeoye		Sonata			
compounds	LRI^a^/LRI^b^	ID	mean±SE		mean±SE		mean±SE		*F*-value	*p*-value
			n = 7		n = 8		n = 7		df = 2, 19	
Total amounts			**53.3±10.5**	***(a)***	**57.6±4.3**	***(a)***	**139.5±26.3**	***(b)***	**11.438**	0,0005
**Aldehydes**										
**Hexanal**	n. d/<1100	A,B^3^	2.5±0.6		2.4±0.1		3.9±0.8		2.457	0,1124
**Heptanal**	908/1198	A,B^5^	1.5±0.4		1.0±0.2		2.1±0.6		2.243	0,1335
**Benzaldehyde**	967/1546	A,B^1^	18.1±3.4		14.5±1.8		20.1±3.4		1.036	0,3741
**Octanal**	1006/1303	A,B^2^	1.2±0.2		1.0±0.1		1.4±0.2		1.747	0,2011
**Nonanal**	1106/1406	A,B^2^	12.7±3.6		11.3±1.3		14.6±2.4		0.447	0,646
**Decanal**	1207/1511	A,B^4^	5.5±1.1		5.4±1.0		6.9±1.3		0.527	0,5986
**p-Anisaldehyde**	1273/2051	A,B^1^	3.9±0.5	*(b)*	2.1±0.4	*(a)*	3.6±0.6	*(b)*	4.320	**0,0284**
**Lily aldehyde**	1535/2063	A,B^5^	0.2±0.0	*(a)*	1.4±0.0	*(b)*	0.3±0.1	*(a)*	3.987	**0,0358**
**Alcohols**										
**1-Hexanol**	<900/	A,B^4^	0.5±0.1	*(a)*	0.8±0.1	*(ab)*	1.1±0.2	*(b)*	6.666	**0,0064**
**(** ***Z*** **)-3-Hexenol**	<900/	A,B^2^	0.6±0.2	*(a)*	1.6±0.2	*(b)*	4.3±1.3	*(c)*	11.398	**0,0006**
**Phenol**	987/2019	A,B^3^	0.6±0.1		1.0±0.5		1.1±0.3		0.784	0,4708
**Benzyl alcohol**	1042/1891	A,B^3^	2.0±0.3	*(a)*	3.8±0.5	*(b)*	4.6±0.5	*(b)*	8.677	**0,0021**
**2-Phenyl ethanol**	1122/1927	A,B^2^	0.3±0.1	*(a)*	0.6±0.1	*(b)*	0.8±0.1	*(b)*	11.009	**0,0007**

Trace indicates average amount less than 0.1 ng g^−1^ flowers. Bold font indicates significant *p-*values for the calculated model (glm). Different lower-case letters indicate significant pairwise differences between respective means of different strawberry varieties at p<0.05 (Tukey test). *: Stereochemistry not determined. Linear retention indices (LRI) were calculated from chromatograms obtained with a HP-5MS (LRI^a^) and an HP-INNOWax (LRI^b^) column. Identification (ID) is based upon mass spectrum matched with those of databases (Wiley 09, Nist 08, and Hochmuth, 2004). LRI is confirmed by synthetic standards. Source of synthetic standards: ^1^ Fluka (Germany), ^2^ Merck-Suchardt (Hohenbrunn, Germany), ^3^ Aldrich (Germany), ^4^ Acros (Germany), ^5^ Sigma-Aldrich (Steinheim, Germany), ^6^ TCI (Zwijndrecht, Belgium). n. d. = non detectable.

**Table 2:** Identified flower volatile compounds of three strawberry varieties (ng g^−1^ flowers). Ester, irregular terpenes and sesquiterpenes.

			Darselect		Honeoye		Sonata			
compounds	LRI^a^/LRI^b^	ID	Mean±SE		Mean±SE		Mean±SE		*F*-value	*P*-value
			n = 7		n = 8		n = 7		df = 2, 19	
**Ester**										
**(** ***Z*** **)-3-Hexenyl acetate**	1010/1327	A,B^3^	0.6±0.2	*(a)*	4.2±1.7	*(b)*	6.2±3,0	*(b)*	19.027	**<0.0001**
**Methyl salicylate**	1201/1803	A,B^5^	0.5±0.1	*(a)*	0.5±0.1	*(a)*	1.5±0,2	*(b)*	27.028	**<0.0001**
**Irregular terpenes**										
**6-Methyl-5-hepten-2-one**	993/1349	A,B^5^	0.1±0.0	*(a)*	0.6±0.1	*(b)*	1.6±0,3	*(c)*	31.324	**<0.0001**
**Dihydro-β-ionone**	1445/1852	A,B^5^	trace	*(a)*	trace	*(a)*	0.2±0,0	*(b)*	21.410	**<0.0001**
**Geranyl acetone**	1457/1867	A,B^1^	0.4±0.1	*(a)*	0.7±0.1	*(b)*	0.8±0,2	*(b)*	2.615	**0,0993**
**β-Ionone**	1494/1955	A,B^5^	0.1±0.0	*(a)*	0.1±0.0	*(a)*	0.3±0,1	*(b)*	9.508	**0,0013**
**Monoterpenes**										
**β-Myrcene**	992/1169	A,B^1^	0.2±0.1	*(a)*	0.2±0.0	*(a)*	0.8±0,1	*(b)*	21.509	**<0.0001**
d/l Limonene^*^	1031/1210	A,B^2^	0.9±0.2	*(ab)*	0.7±0.1	*(a)*	1.4±0,3	*(b)*	2.816	**0,0863**
Ocimene^*^	1039/1259	A,B^1^	0.1±0.0	*(a)*	0.2±0.1	*(a)*	2.6±1,1	*(b)*	12.430	**0,0004**
**Sesquiterpenes**										
**α-Copaene**	1384/1450	A,B^1^	trace	*(a)*	0.2±0.0	*(c)*	0.1±0,0	*(b)*	27.870	**<0.0001**
**(** ***E,E*** **)-α-Farnesene**	1510/1757	A,B^6^	0.8±0.1	*(a)*	4.9±1.6	*(b)*	59.0±17,4	*(c)*	27.778	**<0.0001**

Trace indicates average amount less than 0.1 ng g^−1^ flowers. Bold font indicates significant *p-*values for the calculated model (glm). Different lower-case letters indicate significant pairwise differences between respective means of different strawberry varieties at p<0.05 (Tukey test). *: Stereochemistry not determined. Linear retention indices (LRI) were calculated from chromatograms obtained with a HP-5MS (LRI^a^) and an HP-INNOWax (LRI^b^) column. Identification (ID) is based upon mass spectrum matched with those of databases (Wiley 09, Nist 08, and Hochmuth, 2004). LRI is confirmed by synthetic standards. Source of synthetic standards: ^1^ Fluka (Germany), ^2^ Merck-Suchardt (Hohenbrunn, Germany), ^3^ Aldrich (Germany), ^4^ Acros (Germany), ^5^ Sigma-Aldrich (Steinheim, Germany), ^6^ TCI (Zwijndrecht, Belgium). n. d. = non detectable.

The response of female antenna of *O. bicornis* to volatiles was tested using an electroantennographic setup (EAG) as described in Weissbecker et al. [38]. The tests were carried out by manually injecting the synthetic volatile standards upstream and exposing the dissected antenna into a stream with synthetic air. To guarantee standard conditions, stimuli were supplied every 120 s. Dilutions of synthetic standards in a concentration of 10^−3^ (w/w) were prepared of benzaldehyde, 6-methyl-5-hepten-2-one, (*Z*)-3-hexenyl acetate, d/l-limonene, nonanal, methyl salicylate, p-anidehyde, dihydro-β-ionone, geranyl acetne, β-ionone, and (*E,E*)-α-farnesene, in paraffin oil (Uvasol®, spectrosc. qual., high visc., Merck, Darmstadt, Germany). Approximately 100 µl of standard dilution or paraffin oil, as a control, were dropped on 2 cm^2^ filter paper pieces (Schleicher & Schuell, Dassel, Germany). A piece of soaked filter paper was inserted into a 10 ml glass syringe (Poulten & Graf GmbH, Wertheim, Germany). A typical stimulus was supplied by puffing 5 ml of air over the antenna and repeated once for each compound and control. The EAG response for each compound and control was recorded for *O. bicornis*.

The abundance of females of *O. bicornis* on Sonata and Honeoye was assessed on a commercial strawberry field in the vicinity of Göttingen, Lower Saxony, Germany. The field size was 3.1 ha and both varieties were grown within a distance of 35 m, each on an area of 175 m×35 m in single plant rows, with similar plant quantities per row. Effects of external aspects such as surrounding landscape, field operations, weather conditions, etc were minimized by conducting the field study during simultaneous blooming of both varieties. Two adjacent rows were randomly selected, for each variety, and subdivided into nine transects of 19 m length (along tracks resulting from field operations). Under favourable weather conditions (T>15°C; low cloud cover; wind speed <5 m/s), females of *O. bicornis* foraging on strawberry flowers were counted twice per observation day (morning and afternoon) on each transect and variety, using standardised transect walks of five minutes per transect, at 13 day intervals. Surveys were conducted when both varieties were flowering simultaneously to minimize the influence of other factors such as landscape composition and field operations. For statistical analysis, morning and afternoon samples of all observation days were pooled for each transect.

To analyse differences in the attractiveness between varieties due to morphological variations, we assessed floral display, average flower size and total flower cover. The number of simultaneously blooming flowers was counted to assess the floral display and the size of ten flowers was assessed from three randomly selected plants of each variety on six days during blooming. The average flower size was multiplied by the total number of flowers to calculate the total flower cover per plant for each variety.

Statistical analyses were conducted using the software R, Version 2.13.2 [39]. To test the Differences of the total emission flower volatiles and of distinct compounds among the three varieties were tested using generalized linear models (“glm”-function in package “stats and MASS”) [40] using quasipoisson distribution with variety as fixed factor. Multiple comparisons among varieties were calculated using Tukey contrasts with p-values adjusted by single-step method (“multcomp”-package) [41]. A hierarchical cluster analysis was conducted, using euclidian distance and Ward's methodto determine the general difference between varieties based on the emission of volatile compounds [39]. The difference of EAG responses of *O. bicornis* females among synthetic compounds and paraffin oil and air control were aclculated by fitting generalized linear models using quasipoisson distribution. Differences in the bee abundance between varieties on the commercial strawberry field and the floral display per plant, were tested by fitting generalized linear models with variety as fixed effect. Quasipoisson distribution was used for modelling bee abundance, while negative binomial distribution was required for modelling the number of flowers to account for overdispersion. Linear models with variety as fixed effect were used for calculating differences in the average flower size and overall flower cover between varieties under field conditions. For all analysis, significance was considered at p<0.05.

## Results

Strawberry varieties differed in the overall emission of flower volatile compounds (Table 1, 2). Sonata emitted a significantly higher quantity of flower volatile compounds than Honeoye and Darselect, while emissions from the latter varieties did not differ significantly.

**Figure 1:**
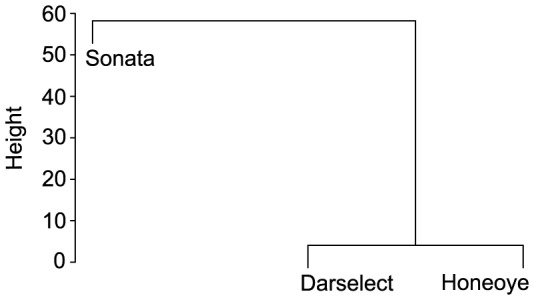
Difference between varieties due to the emission of floral volatile compounds. Differences between varieties are shown as euclidian distance.

In total, strawberry flowers produced 24 different volatile compounds. All three varieties emitted all 24 volatile compounds, but differed in the quantities of several compounds (Table 1, 2). Sonata produced the highest amounts of (*Z*)-3-hexenol, methyl salicylate, 6-methyl-5-hepten-2-one, dihydro-β-ionone, β-ionone, β-myrcene, ocimene and (*E,E*)-α-farnesene. Honeoye and Darselect produced similar quantities of these compounds, except (*Z*)-3-hexenol, 6-methyl-5-hepten-2-one and (*E,E*)-α-farnesene which were higher in Honeoye. Further, Honeoye produced the highest amounts of lily aldehyde and α-copaene. While Darselect was found to produce lower amounts of α-copaene than Sonata, production of lily aldehyde did not differ between these varieties. The emission of benzyl alcohol, 2-phenyl ethanol, (*Z*)-3-hexenyl acetate and geranyl acetone did not differ among Honeoye and Sonata, but all were significantly lower than or differed marginally to Darselect. The latter variety produced intermediate but similar quantities of d/l limonene compared to the other varieties, whereas these differed significantly in the emission of this compound. Similar trends could be observed for the production of 1-Hexanol by Honeoye, which produced this compound in similar but intermediate quantities, whereas Sonata and Darselect differed significantly. The compounds p-anisaldehyde and lily aldehyde were emitted in similar quantities by Darselect and Sonata. While p-anisaldehyde was emitted in lower quantities by Honeoye, lily aldehyde was emitted in higher quantities compared the other varieties. The emission of hexanal, heptanal, benzaldehyde, octanal, nonanal, decanal and phenol did not differ among varieties (Table 1, 2). Hierarchical cluster analysis showed that Honeoye and Darselect differed less in the emission of floral volatile compounds, while the emission of the variety Sonata largely differed compared to both other varieties (Fig. 1)

**Figure 2:**
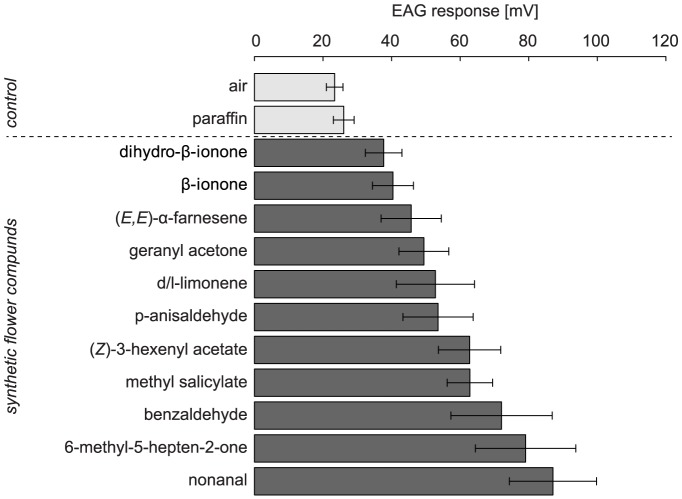
Antennal responses of naïve *O.* *bicornis* females to synthetic compounds. Compounds were identified from flower volatile extracts of strawberry varieties (10^−3^ dilution; mean±SE, n = 10). p<0.05 = significant.

Antennal responses of *O. bicornis* females were significantly higher to all compounds compared with the controls of synthetic air and paraffin oil (Fig. 2). The highest responses were shown on nonanal, 6-methyl-5-hepten-2-one, benzaldehyde, methyl salicylate and (*Z*)-3-hexenyl acetate. Responses to dihydro-β-ionone, β-ionone and (*E,E*)-α-farnesene differed to a lower level, but still significantly from the control treatments, whereas responses to p-anisaldehyde, d/l limonene and geranyl acetone were intermediate.

**Figure 3:**
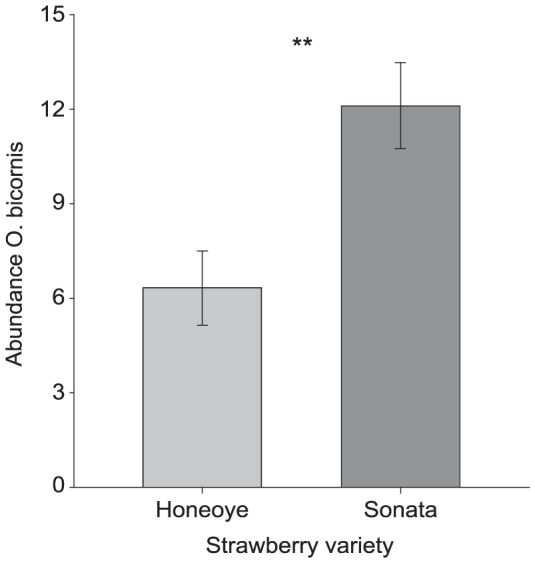
Abundance of *O.* *bicornis* females between strawberry varieties. Data show mean numbers (±SE) of observed specimen per subunit. p<0.05 = significant.

Females of *O. bicornis* were much more abundant flower visitors on Sonata (Fig. 3) compared to Honeoye (F_1,16_ = 11.586; p = 0.004; n = 18). Neither flower display (*Δ*Deviance_1,28_ = 0.934; p = 0.334; n = 18), nor overall flower cover (F_1,28_ = 0.287; p = 0.596; n = 18) differed significantly between varieties under field conditions. The average flower size of Honeoye was significantly greater than flowers of Sonata (F_1,28_ = 16.632; p<0.001; n = 18).

## Discussion

Here we show for the first time, detailed antennal responses of a generalist wild bee pollinator to a broad spectrum of crop flower volatiles and how emission differences between varieties may influence bee visitation rates under field conditions.

The compositions and quantity of the flower volatile compounds emitted by commercially used strawberries have, to date, been reported in only one other study [32]. Hamilton-Kemp et al. studied the volatiles emitted by the variety Redchief and reported surprising differences in composition of volatile compounds compared to varieties that we studied [32]. Almost two thirds of the compounds we found in the current study (hexanal, heptanal, octanal, nonanal, decanal, lily aldehyde, phenol, 6-methyl-5-hepten-2-one, dihydro-β-ionone, geranyl acetone, β-ionone, β-myrcene, α-copaene, (*E,E*)-α-farnesene) were not found by Hamilton-Kemp et al. [32] and thus are reported here for strawberries for the first time. In contrast, we did not find the compounds germacrene D and hexyl acetate in our samples. However volatile emissions and their differences between strawberry varieties have never been reported before.

All compounds emitted by strawberry flowers are known to be generally emitted by flowers [42], [43], [44]. Almost half of the compounds (benzaldehyde, octanal, nonanal, decanal, benzyl alcohol, 2-phenyl ethanol, (*Z*)-3-hexenyl acetate, 6-methyl-5-hepten-2-one, β-myrcene, limonene, ocimene) are found among the most frequently emitted flower volatile compounds [42], [43], [44], [45].

Antennal responses of females of *O. bicornis* to all volatile compounds were higher than responses to controls, though the responses differed among most compounds. Although *Osmia* spp. has been reported to respond to floral scents [28], details about specific compounds are yet to be reported. In contrast, honeybees are known to respond to several of the compounds that were found in the current study, namely (*E,E*)-α-farnesene [7], [46], [47], limonene [46], [48], p-anisaldehyde [49], (*Z*)-3-hexenyl acetate [48], methyl salicylate [48], benzaldehyde [46]. Some of these compounds also seem to evoke responses in certain wild bees. *Bombus terrestris* L. responds to ocimene [50], *Lasioglossum* spp. Curt. to p-anisaldehyde [49] and *Andrena vaga* Panz. to (*E,E*)-α-farnesene [7], [46] and methyl salicylate [7]. However, such detailed responses to various flower volatile compounds as shown in the current study have not been published for solitary wild bees before.

Strawberry varieties differed not only in the overall emission quantity of flower volatile compounds, but also in the quantity of several distinct compounds. In general, differences between Sonata and the other both varieties were larger than between Darselect and Honeoye. From the compounds tested for antennal responses of *O. bicornis*, all compounds were emitted in the highest quantities by Sonata, except nonanal, benzaldehyde, (Z)-3-hexenyl acetate and geranyl acetone. These latter compounds were emitted in similar quantities by Sonata and Honeoye. Darselect emitted all of these compounds in lower quantities compared to the other varieties. These data suggest decreasing responses, which may result in decreasing attractiveness of flowers to bees for Sonata over Honeoye and then lastly Darselect. This finding could be confirmed for Sonata and Honeoye growing in a commercial strawberry field, where females of the most abundant wild bee, *O. bicornis*, visited Sonata much more frequently. Thus bee preference appeared to be related to the emitted volatile compounds [10]. Although females of *O. bicornis* responded to the whole selection of compounds, different concentrations of the same compounds lead to different bee responses. This might support the idea that the relative quantity of certain compounds, creating a unique blend of volatiles, might be a driver for the distinctiveness among floral scents [51], [52]. Different concentrations of distinct volatile compounds have been reported to influence the visitation frequency of honeybees to sunflowers [17] and oilseed rape [19] varieties. However, still practically nothing is known about how the concentration of volatile compounds affects wild bee pollination [9]. Our study could not clarify whether the visitation of *O. bicornis* under field conditions was driven by the distinct blend of volatiles emitted by the variety Sonata or by the higher overall quantity of volatiles emitted. We measured only the antennal responses of bees, which do not necessarily lead to behavioural responses. Additional olfactory experiments are required to test how the behaviour response of bees differs between unique blends and the overall quantity of volatile emissions.

Further, Sonata and Honeoye did not differ in their floral display and flower cover, but flowers of Honeoye were found to be larger than of Sonata. Although larger flowers have been found to be more attractive to bees [53], [54], the abundance of females of *O. bicornis* was higher on Sonata. This supports the hypothesis that floral scents might have been the driver of the higher frequency of visitation of *O. bicornis* females to Sonata under field conditions, and that morphological differences may have been of only minor importance.

The attractiveness of strawberry varieties has further been suggested to differ in relation to the amount and quality of nectar rewards [55]. Although we did not assess these parameters in the current study, these findings seem to be supported by our results as volatile emissions by flowers have been reported to be related to their nectar rewards [7]. These honest signals [9] guide the decision and constancy of pollinators to certain plant species and are thus highly important for plant-pollinator interactions [10].

As shown in our study, certain strawberry varieties are preferred by bees, but all varieties are visited [56], [57]. Sonata and Honeoye did not differ in the emission of Nonanal, Benzaldehye (*Z*)-3-hexenyl-acetate and geranyl acetone. These belong to the most frequently found flower volatile compounds [42], [43], [44], are typical for generalist flowers [45] and are highly attractive for many pollinators [43].

Although (*E,E*)-α-farnesene was produced by Sonata in much higher quantities than all other compounds, antennal responses showed (*E,E*)-α-farnesene belonging to the compounds that were of minor importance for females of *O. bicornis* (dihydro-β-ionone, β-ionone, (*E,E*)-α-farnesene). This compound is, however, known to be highly attractive to honeybees [46], [47]. This may indicate a higher level of specialisation for pollinator species in Sonata compared to Honeoye and Darselect.

Our findings are in line with recent reports on the importance of volatile compounds for the flower selection of *O. bicornis* [11]. Wild bees [58], [59], [60], [61] and especially *Osmia* spp. [23], [26] have been suggested to be major pollinators of crops and may affect the fitness of plants [62].

## Conclusions

Volatile compounds of strawberry flowers appeared to be important in attracting the wild bee *O. bicornis* for sustaining pollination services. To our knowledge, no study has to date shown volatile mediated differences of wild bee attraction between crop varieties, while only three studies showed an influence on honeybee pollination [16], [17], [19]. As varieties of strawberries and other crops differ in the emission of flower volatile compounds, differences in bee visitation rates can be expected to affect pollination success and thereby, yield and quality [7]. Different bee species improve strawberry pollination by complementary behaviour [27]. Hence, the breeding of strawberry varieties, and also the farmer selection of varieties, should focus more closely on flower volatiles, to increase fruit set and thus market value of strawberry fruits via the attraction of a wider range of pollinator species.
