# Correction: Flower Volatiles, Crop Varieties and Bee Responses

**DOI:** 10.1371/annotation/6415e3a5-fed1-4a38-a424-87d860c26db5

**Published:** 2013-09-04

**Authors:** Björn K. Klatt, Carina Burmeister, Catrin Westphal, Teja Tscharntke, Maximilian von Fragstein

There was an error in the name of the fifth author.

The correct name is: Maximilian von Fragstein. 

